# Projecting the Future Registered Nurse Workforce After the COVID-19 Pandemic

**DOI:** 10.1001/jamahealthforum.2023.5389

**Published:** 2024-02-16

**Authors:** David I. Auerbach, Peter I. Buerhaus, Karen Donelan, Douglas O. Staiger

**Affiliations:** 1 Brandeis University, Waltham, Massachusetts; 2 Montana State University, Bozeman, Montana; 3 Institute on Healthcare Systems, Brandeis University, Waltham, Massachusetts; 4 Dartmouth College, Hanover, New Hampshire

## Abstract

**Importance:**

Health care delivery systems rely on a well-prepared and adequately sized registered nurse (RN) workforce. The US RN workforce decreased by more than 100 000 in 2021 during the COVID-19 pandemic—a far greater single-year drop than observed over the past 4 decades. The implication for the longer-term growth of the RN workforce is unknown.

**Objective:**

To describe recent trends in RN employment through 2023 and forecast the growth of the RN workforce through 2035.

**Design, Setting, and Participants:**

Descriptive analysis of recent trends since the start of the COVID-19 pandemic in RN employment using data from the US Bureau of the Census Current Population Survey and including employed RNs aged 23 to 69 years from 1982 through 2023, and retrospective cohort analysis of employment trends by birth year and age to project the age distribution and employment of RNs through 2035.

**Main Outcome and Measures:**

Annual full-time equivalent (FTE) employment of RNs by age, demographics, and sector of employment; forecast of RN workforce by age through 2035.

**Results:**

The final sample included 455 085 RN respondents aged 23 to 69 years. After a sharp decline in 2021, RN employment recovered, and the total number of FTE RNs in 2022 and 2023 was 6% higher than in 2019 (3.35 million vs 3.16 million, respectively). Using data on employment, education, and population through 2022, the size of the RN workforce was projected to increase by roughly 1.2 million FTEs to 4.56 million by 2035, close to prepandemic forecasts. Growth will be driven primarily by RNs aged 35 to 49 years, who are projected to compose nearly half (47%) of the RN workforce in 2035, up from 38% in 2022.

**Conclusions and Relevance:**

In this study, the rebound in the total size of the US RN workforce during 2022 and 2023 indicates that the earlier drop in RN employment during the first 2 years of the COVID-19 pandemic was likely transitory. Updated forecasts of the future RN workforce are very close to those made before the pandemic.

## Key Points

**Question:**

Has the current and projected number of registered nurses (RNs) in the US changed after the COVID-19 pandemic?

**Findings:**

This study found that after a substantial drop during the pandemic, the nursing workforce recovered in 2022 and 2023, and the future size is now expected to reach 4.56 million in 2035, similar to what had been forecast prior to the pandemic.

**Meaning:**

While health care organizations that rely on RNs were challenged with labor shortages during the pandemic, future workforce challenges are less likely to involve an overall shortage than it appeared during the pandemic, and competition for RNs across health care sectors will likely remain robust.

## Teaser

This study describes recent trends in registered nurse (RN) employment through 2023 in the US and forecasts the growth of the RN workforce through 2035.

## Introduction

For decades, health care delivery systems in the US have relied on the steady growth of the registered nurse (RN) workforce. The number of employed RNs (full-time equivalents [FTEs]) in the US nearly tripled from 1.1 million in 1982 to 3.2 million in 2019, an increase from 5.0 to 9.6 RNs per 1000 US residents. However, during the COVID-19 pandemic, the RN workforce has been in flux, and its continued growth is uncertain.^1^ The extensive needs of the first 2 years of the pandemic in 2020 and 2021 placed extraordinary demands on health care workers and organizations, particularly for nurses who were often at the center of this crisis. Shortfalls in RN staffing in elective, acute, and long-term care services were widely reported, as were reports of RN burnout, furloughs, early retirement, and workplace dissatisfaction.^2,3,4^

With these dynamics at play, a prior analysis of national workforce data found that the total number of employed RNs in the US decreased by 100 000 in 2021, the largest single-year reduction since 1980.^5^ This sudden and sharp decrease contributed to concerns about both the adequacy of the current nursing workforce and the future number of RNs available to care for a growing and aging US population.^6^

The primary purpose of this study was to update forecasts of the future number of RNs through 2035. Although such forecasts are inherently uncertain because they rely on key assumptions, they have historically provided information useful for decision-making by educational and health care delivery organizations, along with key policy agencies concerned about the adequacy and sustainability of the workforce. For this analysis, to project the size of the RN workforce through 2035, we applied a labor supply cohort model we have used in prior forecasts.^7,8,9,10,11,12,13,14,15^ Because of the large decrease in the number of employed RNs in 2021, we analyzed data through 2023 to assess whether this reduction was a temporary deviation or represents a more lasting change that could significantly affect our forecast of the total size of the future RN workforce. We combined these data with updated information on entry into nursing schools to construct updated forecasts of the future supply of RNs. To assess the longer-term consequences of the pandemic, we compared these updated forecasts to pre–COVID-19 forecasts of the supply of RNs through 2035.

## Methods

### Data

We obtained monthly data from the Current Population Survey (CPS) for January 1982 through October 2023.^16^ The CPS is administered by the US Census Bureau and is used by the federal government to report monthly unemployment rates, along with demographics, employment status, hours worked, and earnings, yielding monthly samples of between 900 and 1300 RNs. These data are publicly available 1 to 2 months after collection, making the CPS one of the few sources of timely individual-level workforce data before and during the COVID-19 pandemic. Institutional review board approval was not required owing to the use of publicly available, deidentified data.

We constructed estimates of FTE employment by the RNs’ age, survey year, industry setting of employment, gender, marital status, and advanced practice RN (APRN) status. FTE employment was defined as reported usual weekly hours worked divided by 40, summed over all individuals who were working in the week of the survey. Average hours worked and average hourly wage were based on reported usual weekly earnings and usual weekly hours worked. Respondents’ answers regarding their industry setting, ie, hospital, tend to correspond to their work environment rather than ownership structure and concord with estimates based on the far more detailed National Sample Survey of Registered Nurses.^17^ We used sampling weights provided by the CPS to make all estimates nationally representative. Our final sample included 455 085 respondents aged 23 to 69 years who reported working as an RN. We excluded RNs outside of these age ranges due to limited sample sizes in the CPS (these RNs account for 2.3% of all RNs and 2.0% of FTE RNs). APRNs were not separately identified in the CPS prior to 2011 and therefore are combined with RNs in our sample for forecasting purposes.

While data from the CPS are commonly used for analysis of employment and earnings at the occupation level, this data source has some limitations. First, occupation is only identified for those in the labor force and is based on the current or most recent job. Therefore, we had no information on RNs who were working in other occupations or out of the labor force. Second, while response rates in the CPS are generally over 80%, they declined in the early months of the pandemic and ranged from 65% to 75% from March through August 2020.^18^ However, our estimates used the CPS sample weights that adjusted for the varying response rates, which should minimize potential bias.

We also used data on education trends in the nursing workforce from 2000 to 2022 from 2 sources, including the number of completed bachelor-of-science nursing school applications reported by nursing schools in a survey of the American Association of Colleges of Nursing and data on domestic first-time takers and passers of the National Council Licensure Examination (NCLEX) required for nursing licensure reported by the National Council of State Boards of Nursing.^19,20^ Completed applications were adjusted by reported school response rates, which were generally greater than 90%. Finally, additional data on the US population by year and age between 1982 and 2022 were obtained from the US Census Bureau.^21^ Forecasts of the US population through 2035 by age were obtained from the main series projections prepared by the US Census Bureau.^22^

### Statistical Analysis

#### Trends

We used these data to construct national estimates of FTE employment of RNs each year from 1982 to 2023. Data from 2018 to 2023 were used to estimate the change in FTE RNs by industry setting of employment, gender, marital status, and APRN status and to estimate changes in average hours worked and average hourly wage. Because 2023 data were only available through October for this analysis, 2023 data were prorated to represent the full year of 2023 for descriptive analyses. The full forecast model used data only through 2022 because other key data used by the model were only available through 2022. Standard errors and confidence intervals for all estimates were constructed using methods recommended by the US Bureau of Labor Statistics.^23^ Tests of significance and confidence intervals were 2-sided using a 5% level of significance.

#### Model

We forecast the number of FTE RNs by age and year through 2035 using a simple statistical model commonly used by demographers and economists,^24^ applying methods originally described elsewhere in greater detail.^7^ The underlying premise of the model is that individuals initially choose careers in nursing based on the attractiveness of nursing relative to other careers, and, once they become an RN, most continue in the profession for long careers. While the forces that influence career choice have changed across cohorts, life-cycle patterns of work effort have remained relatively similar. Therefore, once we observe production of RN FTEs for a given birth cohort for a few years (for example, individuals born in 1995 who become RNs are observed in our data 5 times, from 2018, at age 23 years, through 2022, at age 27 years), we can predict the future trajectory of FTEs to produce reasonably accurate forecasts. This pattern of a cohort with high numbers of RN FTEs at early ages having high FTEs at later ages has held historically (eFigure 1 in Supplement 1).

The model decomposes observed changes over time in the size and age of the RN workforce into the product of 3 distinct components: population, cohort, and age effects. The population effect captures demographic shifts in the age distribution in the US, such as the aging of the baby boom generation. The cohort effect captures changes in the propensity of individuals born in different years to become RNs as the attractiveness of entering nursing as a career has changed over time. Finally, the age effect captures changes in work patterns over the life cycle as nurses enter the workforce at young ages, have children (in some cases), and then approach retirement. A more technical description of the model is provided in the eMethods in Supplement 1.

#### Estimation

The equation used to estimate cohort effects and age effects is the following:

ln(FTE RN/population) = ln(cohort) + ln(age) + ln(age-cohort interaction).

We used analysis of variance to estimate the parameters of this equation. The dependent variable was the natural logarithm of the number of FTE RNs at every age between 23 and 69 years each year between 1982 and 2022 (47 years of age and 41 years of data on employment equals 1927 total observations) divided by the total US population in that given year-age cell. We defined birth year as survey year minus age, yielding estimates of cohort effects for cohorts born between 1913 and 1999. Based on prior work, in addition to age and cohort effects, we included a set of interaction terms that allow for different age effects below age 30 years for cohorts born after 1964 to capture a secular shift toward later entry into nursing school,^9^ and different age effects above age 50 years for cohorts born after 1940 to capture a secular shift toward delayed retirement.^9^

#### Forecasting

Forecasts of the total number of FTE RNs of each age in 2035 were constructed based on the equation above and summed over all ages to produce aggregate annual forecasts. Population estimates for 2035 were obtained from the US Census main series projections. For cohorts born after 1999 that have not yet entered the labor force by 2022 (and are thus not observed in our data), we use the average cohort effect of the 5 most recently observed cohorts (1995-1999). We further adjusted future cohort size using the recent trend in completed nursing school applications to nursing school based on analysis of data on bachelor of science in nursing (BSN) applicants from the American Association of Colleges of Nursing from 2000 to 2022. On average, a 10% increase in applicants over the most recent 3-year period was associated with incoming RN cohorts that were 3% larger than expected based on the average size of the most recent 5 cohorts.

To assess estimated longer-term impacts of the pandemic, we first used the above data sources only through 2019 to construct a prepandemic forecast of the supply of RNs through 2035. We then compared that prepandemic forecast to an updated forecast based on data through 2022, which captured the impact of the pandemic on the future supply of RNs.

These forecasts assume that age effects observed for previous cohorts will continue in the future and that future cohorts entering the workforce will be comparable in size to recent cohorts (after adjusting for growth in applications). In prior work, we validated this approach using a split-sample method, using data from 1973 to 1988 to forecast the RN workforce a decade later.^8^ The forecast model predicted total workforce growth accurately and correctly predicted that the number of RNs younger than 40 years would decline while the number of RNs 40 years and older would increase over the next 10 years, despite the fact that this trend was not discernible in the data through 1988. We replicate this split-sample validation using nursing workforce data only through 2012 along with the model to forecast the size and age distribution of the nursing workforce in 2022.

Standard errors on forecasts from this model due to sampling error are approximately 5%.^8^ However, the primary sources of uncertainty in the forecasts are the assumptions that age effects will remain stable and that future cohorts will be comparable in size to recent cohorts; our forecast will be too low if nurses increasingly delay retirement in the future or if future cohorts are much larger than recent cohorts. The analysis was performed using Stata, version 17.0 (StataCorp LLC).

## Results

**Figure 1.**
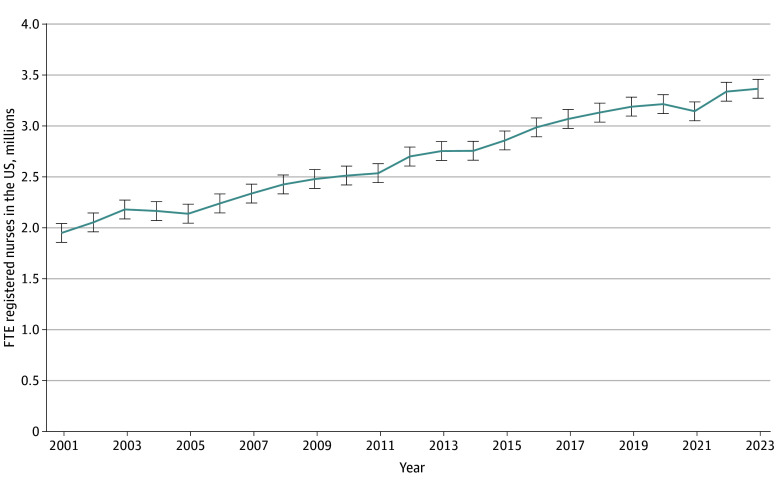
Total Supply of Full-Time Equivalent (FTE) Registered Nurses Through 2023, in Millions Supply is FTEs based on a 40-hour workweek. Error bars represent 95% CIs. Data based on authors’ calculations from the Current Population Survey.^16^

The number of RNs in the workforce grew from just under 2 million FTEs in 2001 to 3.19 million FTEs in 2019 (Figure 1).^16^ After years of steady growth, the size of the RN workforce decreased abruptly by 46 000 in 2020 and 2021 but then swiftly rebounded, growing by 222 000 (95% CI, 47 000-397 000) FTE RNs between 2021 and 2023. By 2023, the number of FTE RNs reached 3.37 million (95% CI, 3.25-3.49 million), 6% higher than just before the pandemic in 2018 and 2019.

Workforce growth from 2018-2019 to 2022-2023 occurred among all age groups but was led by RNs younger than 35 years (8.2% growth), who grew in number at twice the rate of RNs older than 50 years (3.5%; Table). Growth was also more pronounced for male RNs (14.1%), unmarried RNs (7.4%), APRNs (18.2%), and RNs working outside of hospital settings (12.8%). The shift in RN employment away from hospitals (the percentage employed in hospitals dropped from 60.3% before the pandemic to 57.8% after) was entirely due to a drop in hospital employment among RNs older than 40 years.

**Table.** Characteristics of the US Registered Nurse (RN) Workforce in 2018-2019 and 2022-2023^a^

Characteristic	No. (% of all RNs)		Change, 2018-2019 to 2022-2023, No.	% Change (95% CI)
	2018-2019	2022-2023		
All RNs (FTE)	3 163 072 (100)	3 353 552 (100)	190 481	6.0 (1.4 to 10.6)
Age, y				
<35	944 247 (29.9)	1 021 333 (30.5)	77 085	8.2 (−0.3 to 16.7)
35-49	1 189 695 (37.6)	1 267 073 (37.8)	77 378	6.5 (−1.0 to 14.0)
≥50	1 029 129 (32.5)	1 065 147 (31.8)	36 018	3.5 (−4.5 to 11.5)
Gender				
Men	389 235 (12.3)	443 968 (13.2)	54 734	14.1 (0.6 to 27.5)
Women	2 773 837 (87.7)	2 909 584 (86.8)	135 747	4.9 (0.0 to 9.8)
Marital status				
Married	1 920 804 (60.7)	2 019 736 (60.2)	98 932	5.2 (−0.8 to 11.1)
Nonmarried	1 242 268 (39.3)	1 333 817 (39.8)	91 549	7.4 (0.0 to 14.8)
Role				
Non-APRN	2 918 885 (92.3)	3 064 955 (91.4)	146 069	5.0 (0.2 to 9.8)
APRN	244 186 (7.7)	288 598 (8.6)	44 411	18.2 (1.1 to 35.3)
Work setting				
Hospitals				
All RNs	1 908 177 (60.3)	1 938 235 (57.8)	30 058	1.6 (−4.3 to 7.4)
RNs aged <40 y	876 997	957 613	80 616	9.2 (0.4 to 18.0)
RNs aged ≥40 y	1 031 180	980 621	−50 558	−4.9 (−12.8 to 3.0)
All other settings	1 254 895 (39.7)	1 415 318 (42.2)	160 423	12.8 (5.3 to 20.2)
Average hours worked	37.3	37.5	0.2	0.6 (−2.10 to 3.30)
Average hourly wage, $^b^	32.36	37.46	5.10	15.8 (13.10 to 18.50)

Abbreviations: APRN, advanced practice registered nurse; FTE, full-time equivalent.

^a^ Data shown are averages of the 2 years indicated. The 2023 data include data through October and are prorated to represent a full year.

^b^ Wages are reported in the years shown (are not inflation adjusted).

**Figure 2.**
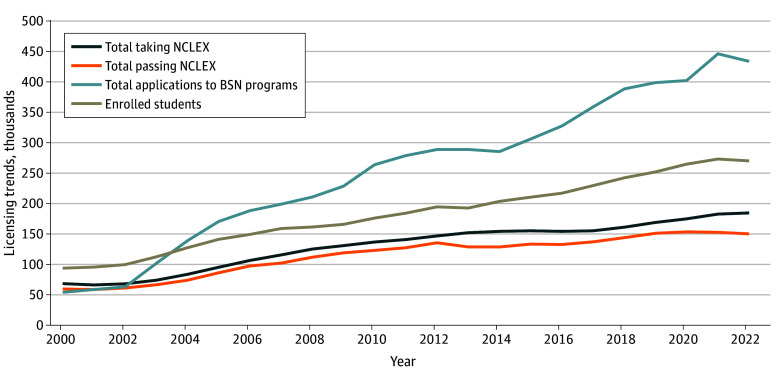
Trends in Nursing Education (in Thousands), 2000-2022 Data based on National Council of State Boards of Nursing and American Association of Colleges of Nursing.^19^ BSN indicates bachelor of science in nursing; NCLEX, National Council Licensing Examination.

Growth in entry into nursing among younger RNs is also evidenced by trends in education from 2000 to 2022 (Figure 2).^19^ The number of completed applications to BSN programs and enrolled students in such programs have grown substantially over the past 20 years, though both dipped slightly in 2022. The number of US domestically trained students taking the NCLEX for the first time has also increased since 2000 and substantially from 2016 to 2021 (from 154 000 to 185 000), although NCLEX pass rates dropped in the past several years (from 88% in 2019 to 80% in 2022), leaving the number passing the examination relatively constant from 2019 to 2022.

### Forecasting the Future RN Workforce

Using data from 1982 to 2022, we estimated age and cohort effects as described above and then used these estimates to form forecasts of RN FTE employment through 2035. Both age and cohort estimated effects were significant (*P* < .001, supporting data in the eTable in Supplement 1), and the model was fit with an *R*^2^ of 0.9298. The age estimated effects for recent birth cohorts at each age relative to age 45 years (eFigure 2 in Supplement 1) describe the life-cycle pattern of production of RN FTE work for an average birth cohort. FTEs for a given birth cohort tend to peak in the late 40s, with roughly twice the FTE production as at age 26 years, for example.

The cohort estimated effect for birth cohorts from 1930 to 1995 (eFigure 3 in Supplement 1) describes the amount of entry into nursing for those born in each year using the estimated total number of nursing FTEs produced when each birth cohort was age 40 years. The figure shows a relative peak with the 1955 cohort (who reached age 40 years in 1995), followed by a small decline over the next 10 to 15 years, and then rapid growth among those born from 1981 to 1996, far outpacing earlier entrants into the nursing workforce.

To validate the model, we conducted a split-sample forecast, populating the model with nursing workforce data only through 2012 and forecasting the size and age distribution of the nursing workforce in 2022, which we then compared to the actual observed workforce in 2022. As reported in eFigure 4 in Supplement 1, the forecast accurately predicted 10 years into the future that the number of RNs in their 30s would grow substantially (forecast growth, 38%; actual growth, 46%) and that the number of RNs in their 50s would decline (forecast growth, −12%; actual growth, −6%). The overall workforce grew faster than projected, largely due to greater entry into nursing education than anticipated and greater workforce output among RNs in their 60s than anticipated.

By 2035, the final forecast year, the number of FTE RNs is expected to reach over 4.5 million RNs (4 556 000), an increase of 36% from 3.3 million as of the end of 2022 (Figure 1). This forecast is slightly below our prepandemic forecast of 4.64 million but above what our forecast would have been using data only through the end of 2021 (4.46 million) and assuming that the COVID-19–related reduction in supply in 2020 and 2021 would persist. Thus, the recovery of RN employment in 2022 contributed meaningfully to our forecast, and 2023 data through October further confirm that the 2022 recovery is robust.

**Figure 3.**
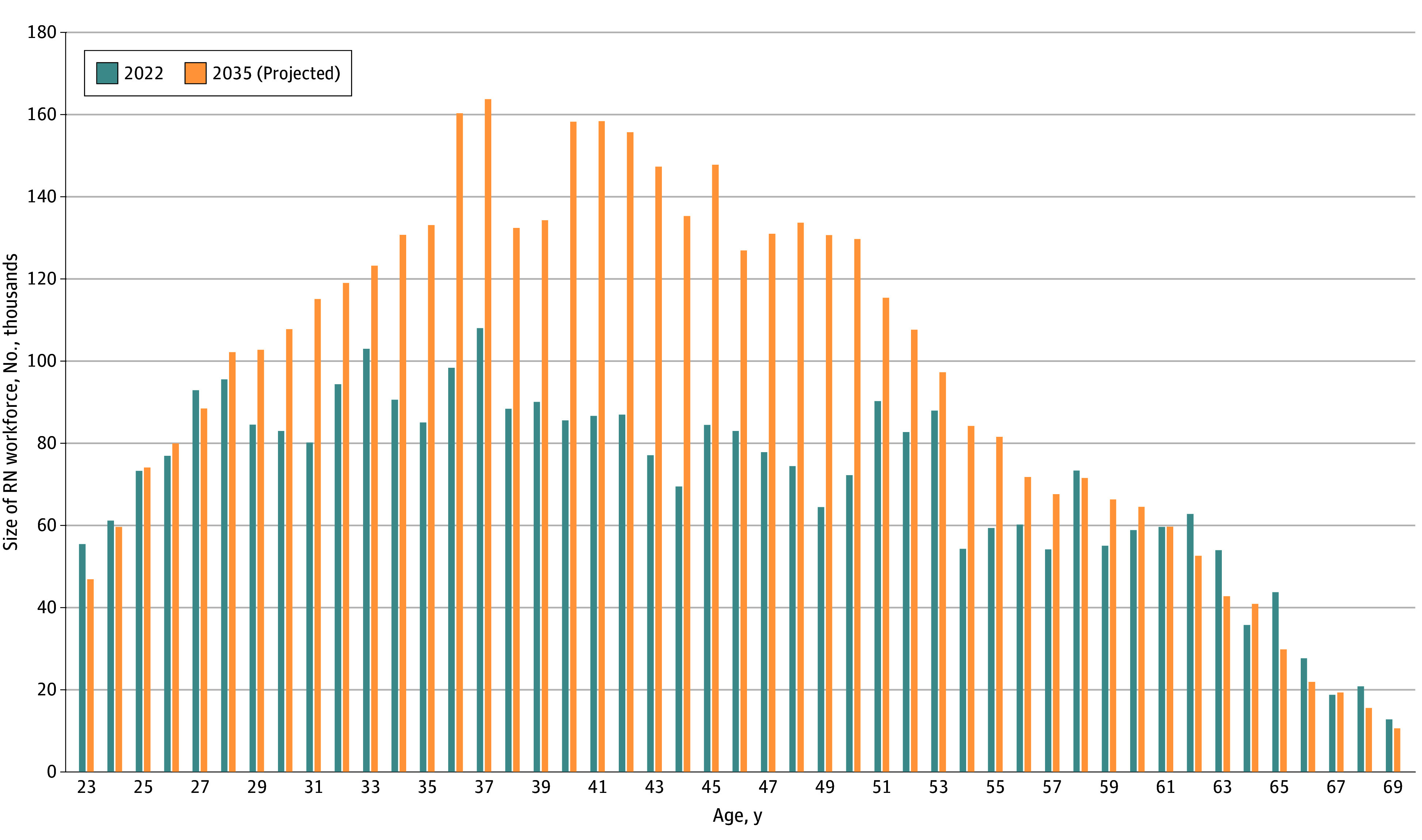
Total Current and Projected Size of the Registered Nurse (RN) Workforce by Age, 2022 and 2035 RNs younger than 23 years or older than 70 years were excluded. Data represent full-time equivalents.

Model results indicate that workforce growth will be driven predominantly by RNs aged 35 to 49 years, who are projected to increase in number by nearly 900 000 RNs over this time (from 1.3 million RNs to 2.2 million RNs), increasing their share of the nursing workforce from 38% in 2022 to 47% in 2035 (Figure 3). This growth accounts for 73% of the total forecast workforce growth of 1.2 million RNs over this time. Meanwhile, RNs older than 50 years are projected to decrease as a proportion of all RNs from 33% in 2022 to 27% in 2035.

## Discussion

Our forecast of the US RN workforce indicates that the national supply of employed RNs will grow substantially through 2035. The forecasted growth will be large enough to replace RNs who will retire and further expand the workforce by RNs by roughly 1.2 million by 2035. The makeup of the RN workforce by age will shift toward RNs aged 35 to 49 years, who represent 38% of RNs in 2023 but will account for nearly half of all RNs in 2035. Overall, this forecast suggests that the pandemic’s impact on employed RNs, at least thus far, is unlikely to have a significant impact on the future growth of the overall RN workforce.

Several uncertainties should be considered. First, factors affecting the inflow to nursing education have been changing. The number of applications to BSN programs has risen rapidly over the past 20 years (more than doubling), suggesting continued interest in nursing careers. Yet the pandemic likely decreased the academic preparedness of some high school and college students, which could slow their educational progression, test-taking readiness, and eventual entry into the RN workforce.^25^ There was a sharp decline in NCLEX pass rates during the pandemic (although test scores have rebounded as of mid-2023 with the introduction of a major test redesign).^26^ Nursing education programs and employers should consider jointly assessing how adequately prepared new nursing graduates are to begin practicing. Should deficits be identified, educators and employers can bolster existing mentorship and onboarding programs to address gaps in education and clinical preparation.

Second, there is uncertainty about the future demand for RN labor. Many hospitals and health systems are testing new models of care amid staffing challenges. Such innovation might lessen or increase the demand for RNs.

Finally, our study suggests possible shifts in where RNs work. Workforce growth from 2018 to 2023 occurred almost entirely in nonhospital settings and may reflect a shift of RN employment away from hospitals and into ambulatory and community settings. This shift may help explain why some hospitals have reported shortages of RNs, despite robust growth of the overall workforce in 2022 and 2023.

### Limitations

Our forecast model rests on underlying assumptions that interest in nursing careers, entrance into nursing, and nursing retirement patterns will remain relatively steady over the next decade. Major disruptions, or factors we have not measured, could lead the forecast to prove inaccurate. Our analysis is further limited by the lack of available data on APRNs prior to 2011, when RNs and APRNs were not distinguished in our main data source. We have explored the impact of that limitation on our modeling and expect that the forecast would not change substantially as a result of this exclusion.

## Conclusions

This study found that the US nursing workforce recovered in 2022 and 2023, and the future size is estimated to reach 4.56 million in 2035, similar to what had been forecast prior to the pandemic. We expect continued robust growth in the US RN workforce, largely due to the strong and sustained growth of RNs who are now in their late 20s and 30s. Whether this forecasted growth will satisfy needs for the types of health care services provided by RNs, or match health care delivery organizations’ demand for RN labor, remains to be seen. These uncertainties suggest a heightened need to continue to monitor changes in the US RN workforce.

## Notes

Supplement 1. **eFigure 1.** Average US Registered Nurses (FTEs) produced by age for selected 5-year birth cohorts **eMethods.** Model description **eTable.** Regression output from the model estimation regression equation **eFigure 2.** US Registered Nurse FTE production by age relative to age 45 **eFigure 3.** RN FTEs at age 40 by cohort year of birth **eFigure 4.** Observed United States FTE RNs by age group in 2012, forecast FTE RNs by age group in 2022 based on data only through 2012 and observed FTE RNs by age group in 2022

Supplement 2.
Data Sharing Statement
