# Supplementary material for: Projecting the Future Registered Nurse Workforce After the COVID-19 Pandemic
Source: JAMA Health Forum. 2024 Feb 16;5(2):e235389. doi: 10.1001/jamahealthforum.2023.5389 (PMC10873770; doi:10.1001/jamahealthforum.2023.5389)
Supplement: Supplement 1. — eFigure 1. Average US Registered Nurses (FTEs) produced by age for selected 5-year birth cohorts eMethods. Model description eTable. Regression output from the model estimation regression equation eFigure 2. US Registered Nurse FTE production by age relative to age 45 eFigure 3. RN FTEs at age 40 by cohort year of birth eFigure 4. Observed United States FTE RNs by age group in 2012, forecast FTE RNs by age group in 2022 based on data only through 2012 and observed FTE RNs by age group in 2022 [file jamahealthforum-e235389-s001.pdf]

Supplemental Online Content

Auerbach DI, Buerhaus PI, Donelan K, Staiger DO. Projecting the future registered nurse workforce after the COVID-19 pandemic. *JAMA Health Forum.* Published online February 16, 2024. doi:10.1001/jamahealthforum.2023.5389

eFigure 1. Average US Registered Nurses (FTEs) produced by age for selected 5-year birth cohorts

eMethods. Model description

eTable. Regression output from the model estimation regression equation

eFigure 2. US Registered Nurse FTE production by age relative to age 45

eFigure 3. RN FTEs at age 40 by cohort year of birth

eFigure 4. Observed United States FTE RNs by age group in 2012, forecast FTE RNs by age group in 2022 based on data only through 2012 and observed FTE RNs by age group in 2022

This supplemental material has been provided by the authors to give readers additional information about their work.

© 2024 Auerbach DI et al. *JAMA Health Forum.*

Supplemental Materials

eFigure 1. Average US Registered Nurses (FTEs) produced by age for selected 5-year birth cohorts

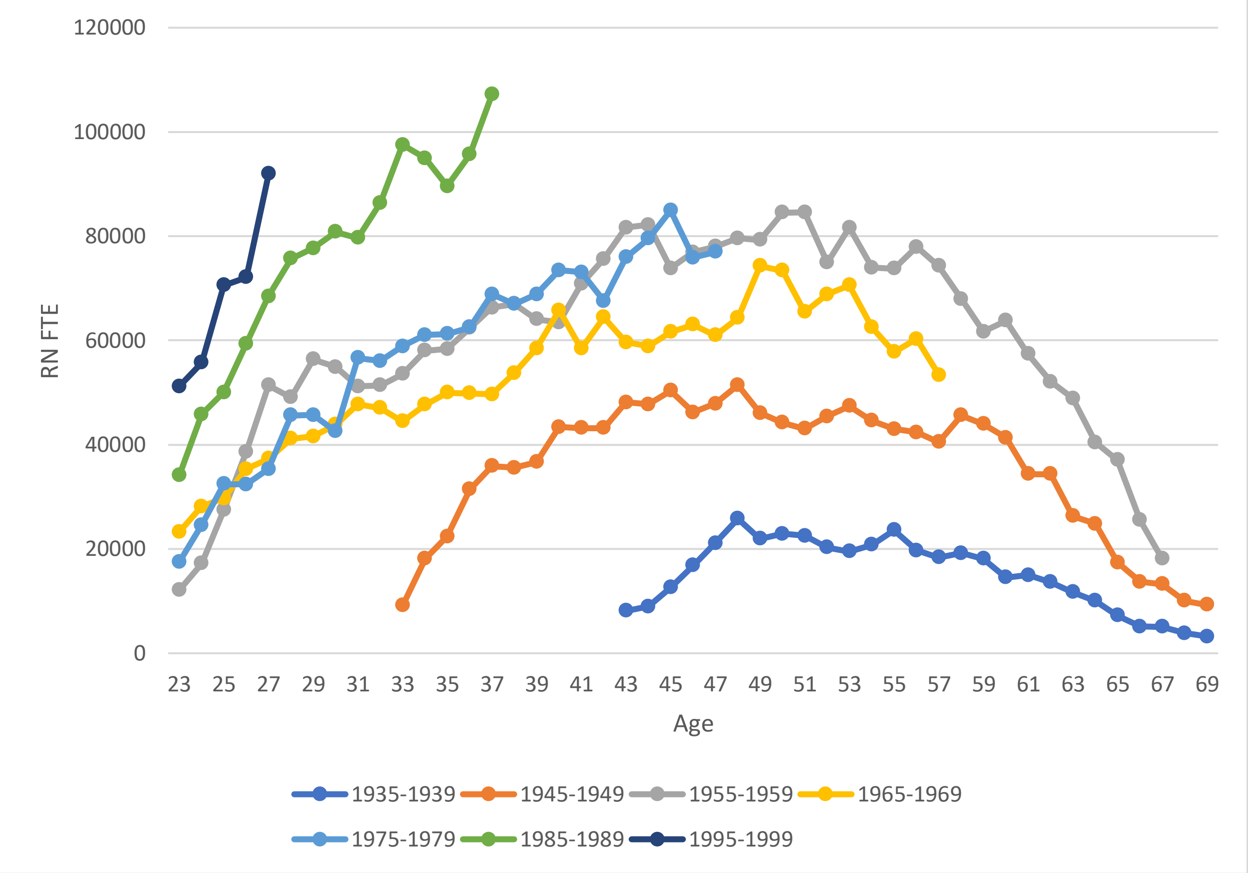

Notes: Data points represent the average number of FTE RNs observed at the age shown among the 5 birth cohorts as indicated in the legend.
For example, there were an average of 79,579 RN FTE observed at age 48 among RNs born between 1955 and 1959, which were observed in the years 2003 through 2007.
Data Source: U.S. Current Population Survey

For example, the 1955-1959 birth cohorts yielded roughly 50% more RNs at every age than the 1945-1949 birth cohorts while the shape of their career arcs, as members of the birth cohorts entered nursing school, took time off to raise children (in some cases), and ultimately retired in their 60s, remained similar.

© 2024 Auerbach DI et al. *JAMA Health Forum.*

eMethods. Model description

The dependent variable is the number of FTE RNs of a given single year of age (a) that were born in a given year (b) (for example, the observed number of observed FTEs produced by RNs of age 30 in 2015) is described by the following equation:

(1) No. of FTE $RN_{a,b}$ = ($POPULATION_{a,b}$)(δ_b)(α_a)

The first term on the right hand side of equation 1 is the population effect, with $POPULATION_{a,b}$ referring to the total US population of a given birth cohort (b) at a given age (a). The second term (δ_b) is the cohort effect, representing the relative propensity of individuals in birth cohort b to work as RNs. The final term (α_a) is the age effect, representing the relative propensity of RNs to work at each age (a).

Based on prior work, in addition to age and cohort effects, we included two sets of interaction terms (denoted $\alpha_{a,b}$ in equation 2 below) that accounted for: (1) different age effects below age 30 for cohorts born after 1964 to capture a secular shift toward later entry into nursing school (interacting a dummy for being in a cohort born after 1964 with dummies for age 23-24, 25-27, and 28-29), and (2) different age effects above age 50 for cohorts born after 1940 to capture a secular shift toward delayed retirement (interacting a dummy for being in a cohort born after 1950 with each age dummy above age 50).

To estimate the model, we take the natural log of both sides to yield:

(2) ln (No. of FTE $RN_{a,b}$ / $POPULATION_{a,b}$) = ln(δ_b) + ln(α_a) + ln ($\alpha_{a,b}$)

We used Analysis of Variance (ANOVA) to estimate the parameters of this equation, and exponentiated the resulting cohort and age intercepts (adding the interaction terms for the

© 2024 Auerbach DI et al. *JAMA Health Forum.*

indicated cohorts) to yield estimates of δ_b and α_a. The dependent variable was the logarithm of the number of FTE RNs at every age between 23 and 69 for every year between 1982 and 2022 (47 years of age and 41 years of data on employment equals 1,927 total observations) divided by the total US population in that given year-age cell.

Model results

eTable. Regression output from the model estimation regression equation

Dependent variable is the log of RN FTEs per 100,000 population in a given age-year cell. Coefficients can be interpreted as the percentage difference in RNs in that age-year cell given the variable value relative to a reference group, e.g. 22.9% more FTEs expected at age 24 than at age 23 (the omitted age).

Definitions of variables:

Dependent variable: “lnpopfte” is the natural log of the number of FTEs per capita provided by a given single-year birth cohort at a given age in a given year.

Age = dummy variables for single year of age for the RN. Age 23 is omitted.
ybirth = year of birth for RN. 1913 is omitted.

Age-cohort interaction terms:
z1 = age 28-29 and born after 1964
z2 = age 25-27 and born after 1964
z2 = age 23-24 and born after 1964
a1 = age = 50 and birth year>1940
a2 = age = 51 and birth year>1940
….
a19 = age = 69 and birth year >1940

Variable	Coefficient	Std. err.	t	P>t	[95% conf.	interval]
lnpopfte						
z						
1	-.1238604	.0565471	-2.19	0.029	-.2347665	-.0129543
2	-.2460696	.0513669	-4.79	0.000	-.3468157	-.1453234
3	-.4984206	.0695202	-7.17	0.000	-.6347709	-.3620702
age						
24	.2291872	.0484409	4.73	0.000	.1341799	.3241945
25	.1832598	.083624	2.19	0.029	.0192476	.3472719
26	.248331	.0831045	2.99	0.003	.0853377	.4113243
27	.3385348	.0826226	4.10	0.000	.1764866	.500583
28	.3567385	.0842703	4.23	0.000	.1914587	.5220182

© 2024 Auerbach DI et al. *JAMA Health Forum.*

29	.3590862	.0836875	4.29	0.000	.1949494	.523223
30	.2788233	.0757009	3.68	0.000	.1303507	.4272959
31	.3411929	.0756434	4.51	0.000	.1928331	.4895527
32	.3709623	.0755933	4.91	0.000	.2227009	.5192238
33	.402865	.0755494	5.33	0.000	.2546896	.5510404
34	.4596044	.0755109	6.09	0.000	.3115044	.6077043
35	.4749607	.0754773	6.29	0.000	.3269267	.6229946
36	.4911565	.0754479	6.51	0.000	.3431801	.6391329
37	.5230883	.0754225	6.94	0.000	.3751618	.6710148
38	.5426823	.0754007	7.20	0.000	.3947986	.6905661
39	.5606336	.0753823	7.44	0.000	.412786	.7084813
40	.6621616	.0753671	8.79	0.000	.5143437	.8099794
41	.6657475	.0753549	8.83	0.000	.5179535	.8135415
42	.6737586	.0753474	8.94	0.000	.5259794	.8215378
43	.7101988	.0753486	9.43	0.000	.5624171	.8579804
44	.7100879	.0753592	9.42	0.000	.5622856	.8578903
45	.7202393	.0753797	9.55	0.000	.5723967	.8680819
46	.7258354	.0754108	9.63	0.000	.5779317	.873739
47	.7222088	.0754534	9.57	0.000	.5742217	.8701959
48	.8032805	.0755082	10.64	0.000	.6551858	.9513753
49	.7516695	.0755764	9.95	0.000	.6034412	.8998979
50	.7735327	.0756589	10.22	0.000	.6251425	.9219229
51	.7413578	.1029381	7.20	0.000	.5394647	.9432508
52	.702054	.1009395	6.96	0.000	.5040808	.9000272
53	.7304046	.0992492	7.36	0.000	.5357465	.9250626
54	.7247669	.0978019	7.41	0.000	.5329475	.9165862
55	.7913149	.0965497	8.20	0.000	.6019516	.9806783
56	.7154299	.0954568	7.49	0.000	.52821	.9026497
57	.7005863	.0945024	7.41	0.000	.5152382	.8859343
58	.6755768	.0936516	7.21	0.000	.4918975	.8592561
59	.6010418	.092895	6.47	0.000	.4188463	.7832373
60	.5348264	.0922197	5.80	0.000	.3539555	.7156974
61	.4419125	.0916152	4.82	0.000	.2622272	.6215978
62	.1703478	.0910843	1.87	0.062	-.0082963	.3489919
63	.0480596	.090598	0.53	0.596	-.1296308	.2257499
64	-.0571817	.0901639	-0.63	0.526	-.2340207	.1196573
65	-.4309376	.08978	-4.80	0.000	-.6070236	-.2548515
66	-.8134929	.0894473	-9.09	0.000	-.9889263	-.6380595
67	-1.218781	.0895426	-13.61	0.000	-1.394402	-1.043161
68	-1.072805	.0893797	-12.00	0.000	-1.248106	-.8975046
69	-1.232328	.0889827	-13.85	0.000	-1.40685	-1.057805
**a**						
1	.0158173	.0873253	0.18	0.856	-.1554542	.1870888
2	.0423122	.0852724	0.50	0.620	-.1249329	.2095573
3	.0290467	.0836051	0.35	0.728	-.1349284	.1930219
4	-.0405937	.0822527	-0.49	0.622	-.2019163	.120729
5	-.1184455	.0811638	-1.46	0.145	-.2776325	.0407415
6	-.0307066	.0803008	-0.38	0.702	-.1882008	.1267877
7	-.0497671	.0796435	-0.62	0.532	-.2059723	.1064381
8	-.0192768	.0791553	-0.24	0.808	-.1745244	.1359708
9	.0448055	.0788239	0.57	0.570	-.1097922	.1994033
10	.0626014	.0786454	0.80	0.426	-.0916462	.216849
11	.077904	.078614	0.99	0.322	-.076282	.2320901
12	.2516956	.0787404	3.20	0.001	.0972615	.4061296
13	.2039675	.0789999	2.58	0.010	.0490246	.3589105
14	.2206047	.0794122	2.78	0.006	.0648533	.3763562
15	.3396014	.0799869	4.25	0.000	.1827227	.4964801
16	.4790072	.0807405	5.93	0.000	.3206504	.637364
17	.8050689	.0821033	9.81	0.000	.6440393	.9660984
18	.4720887	.0833415	5.66	0.000	.3086305	.6355469
19	.3035296	.0845276	3.59	0.000	.1377452	.4693139
ybirth						
1914	.9079472	.2684834	3.38	0.001	.3813693	1.434525
1915	.228452	.2539226	0.90	0.368	-.2695677	.7264717

© 2024 Auerbach DI et al. *JAMA Health Forum.*

1916	.0514316	.2463173	0.21	0.835	-.4316718	.5345349
1917	.5412144	.2416495	2.24	0.025	.0672659	1.015163
1918	.3612872	.2384939	1.51	0.130	-.1064722	.8290466
1919	.0929686	.2362193	0.39	0.694	-.3703296	.5562669
1920	.7411593	.236257	3.14	0.002	.2777873	1.204531
1921	.9426457	.2331637	4.04	0.000	.4853405	1.399951
1922	1.073443	.2320897	4.63	0.000	.6182441	1.528642
1923	.9511734	.23121	4.11	0.000	.4977	1.404647
1924	1.16771	.230477	5.07	0.000	.7156741	1.619745
1925	1.195048	.2305334	5.18	0.000	.7429015	1.647194
1926	1.182716	.2293273	5.16	0.000	.7329347	1.632496
1927	1.123924	.2288693	4.91	0.000	.6750418	1.572807
1928	1.213645	.2284702	5.31	0.000	.7655449	1.661745
1929	1.262031	.2281199	5.53	0.000	.8146177	1.709443
1930	1.283326	.2278107	5.63	0.000	.8365198	1.730132
1931	1.541673	.2275364	6.78	0.000	1.095404	1.987941
1932	1.602775	.2272742	7.05	0.000	1.157021	2.048529
1933	1.431795	.2270468	6.31	0.000	.9864868	1.877103
1934	1.47868	.2268493	6.52	0.000	1.033759	1.9236
1935	1.754746	.2266771	7.74	0.000	1.310163	2.199329
1936	1.598447	.2265269	7.06	0.000	1.154158	2.042735
1937	1.726025	.2263957	7.62	0.000	1.281993	2.170056
1938	1.804858	.2262811	7.98	0.000	1.361052	2.248665
1939	1.646009	.2261811	7.28	0.000	1.202399	2.089619
1940	1.790137	.2260938	7.92	0.000	1.346698	2.233576
1941	1.813705	.2291029	7.92	0.000	1.364364	2.263046
1942	1.797017	.228982	7.85	0.000	1.347913	2.246121
1943	1.915079	.228869	8.37	0.000	1.466197	2.363961
1944	1.826089	.2287632	7.98	0.000	1.377415	2.274764
1945	1.947149	.2286639	8.52	0.000	1.498669	2.395629
1946	1.930703	.2285706	8.45	0.000	1.482407	2.379
1947	2.044798	.2284827	8.95	0.000	1.596674	2.492922
1948	2.037854	.2283998	8.92	0.000	1.589892	2.485816
1949	2.053035	.2283216	8.99	0.000	1.605226	2.500843
1950	2.112145	.2282477	9.25	0.000	1.664482	2.559809
1951	2.205383	.2281777	9.67	0.000	1.757857	2.652909
1952	2.264611	.2281114	9.93	0.000	1.817215	2.712007
1953	2.307408	.2280502	10.12	0.000	1.860132	2.754684
1954	2.36485	.2280525	10.37	0.000	1.917569	2.81213
1955	2.353026	.2280513	10.32	0.000	1.905748	2.800304
1956	2.34663	.2280545	10.29	0.000	1.899345	2.793914
1957	2.349715	.2280625	10.30	0.000	1.902415	2.797015
1958	2.242873	.228066	9.83	0.000	1.795566	2.690179
1959	2.205006	.2280785	9.67	0.000	1.757675	2.652338
1960	2.209902	.2281415	9.69	0.000	1.762447	2.657357
1961	2.207697	.2282078	9.67	0.000	1.760112	2.655282
1962	2.179132	.2282778	9.55	0.000	1.73141	2.626855
1963	2.104463	.2283516	9.22	0.000	1.656596	2.55233
1964	2.184083	.2284296	9.56	0.000	1.736063	2.632104
1965	2.185612	.2284854	9.57	0.000	1.737482	2.633741
1966	2.127566	.2285728	9.31	0.000	1.679264	2.575867
1967	2.184877	.2286657	9.55	0.000	1.736394	2.633361
1968	2.217203	.2287645	9.69	0.000	1.768527	2.66588
1969	2.258135	.2288698	9.87	0.000	1.809251	2.707018
1970	2.322299	.2289821	10.14	0.000	1.873195	2.771403
1971	2.379308	.2291024	10.39	0.000	1.929968	2.828647
1972	2.332995	.2292313	10.18	0.000	1.883403	2.782588
1973	2.36608	.2294158	10.31	0.000	1.916125	2.816034
1974	2.385641	.2296085	10.39	0.000	1.935309	2.835974
1975	2.377767	.2298107	10.35	0.000	1.927038	2.828495
1976	2.347581	.2300237	10.21	0.000	1.896435	2.798728
1977	2.404237	.2302489	10.44	0.000	1.952648	2.855825
1978	2.345336	.2304881	10.18	0.000	1.893278	2.797393
1979	2.351267	.2307437	10.19	0.000	1.898708	2.803826
1980	2.472231	.231018	10.70	0.000	2.019134	2.925328
1981	2.473724	.2313142	10.69	0.000	2.020046	2.927402

© 2024 Auerbach DI et al. *JAMA Health Forum.*

1982	2.524184	.2316362	10.90	0.000	2.069875	2.978493
1983	2.622464	.231995	11.30	0.000	2.167451	3.077477
1984	2.66459	.2323975	11.47	0.000	2.208788	3.120392
1985	2.749807	.2328522	11.81	0.000	2.293113	3.206501
1986	2.764612	.2333701	11.85	0.000	2.306902	3.222322
1987	2.721224	.2339656	11.63	0.000	2.262346	3.180102
1988	2.767831	.2346577	11.80	0.000	2.307596	3.228066
1989	2.722566	.2354723	11.56	0.000	2.260733	3.184399
1990	2.869921	.2364456	12.14	0.000	2.406179	3.333663
1991	2.782115	.2376292	11.71	0.000	2.316051	3.248178
1992	2.857294	.2391004	11.95	0.000	2.388345	3.326242
1993	2.938861	.2409795	12.20	0.000	2.466226	3.411495
1994	2.971042	.2433794	12.21	0.000	2.493701	3.448383
1995	2.98045	.2467279	12.08	0.000	2.496542	3.464359
1996	2.924112	.2516132	11.62	0.000	2.430622	3.417602
1997	2.935338	.2595746	11.31	0.000	2.426233	3.444443
1998	3.173991	.2748666	11.55	0.000	2.634894	3.713088
1999	3.187625	.3160174	10.09	0.000	2.567818	3.807431
_cons	-7.069477	.2353197	-30.04	0.000	-7.531011	-6.607943

eFigure 2. US Registered Nurse FTE production by age relative to age 45

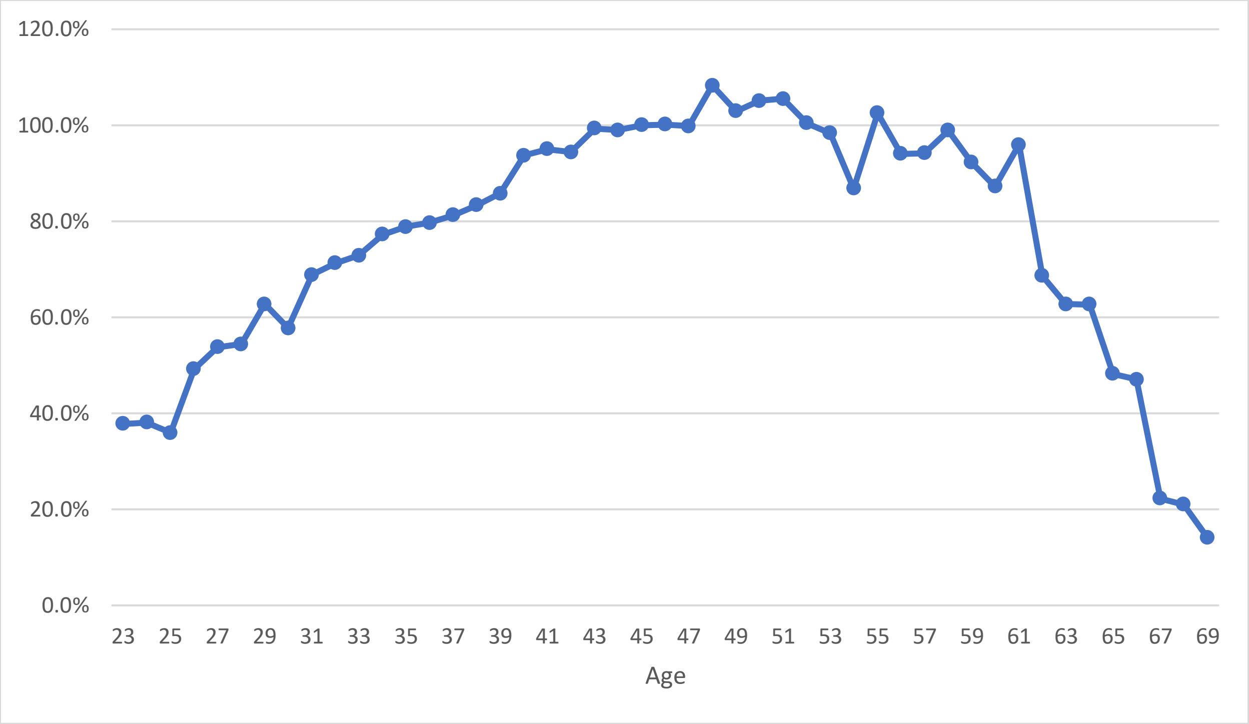

Notes: Data points represent the relative number of RN FTEs produced at every age shown for an average birth cohort born after 1965. For example, among all people born in 1970, there were approximately 80% as many RN FTEs observed when that group was age 36 (in 2006) as when that group was age 45 (in 2015).
Data Source: U.S. Current Population Survey

© 2024 Auerbach DI et al. *JAMA Health Forum.*

eFigure 3. RN FTEs at age 40 by cohort year of birth

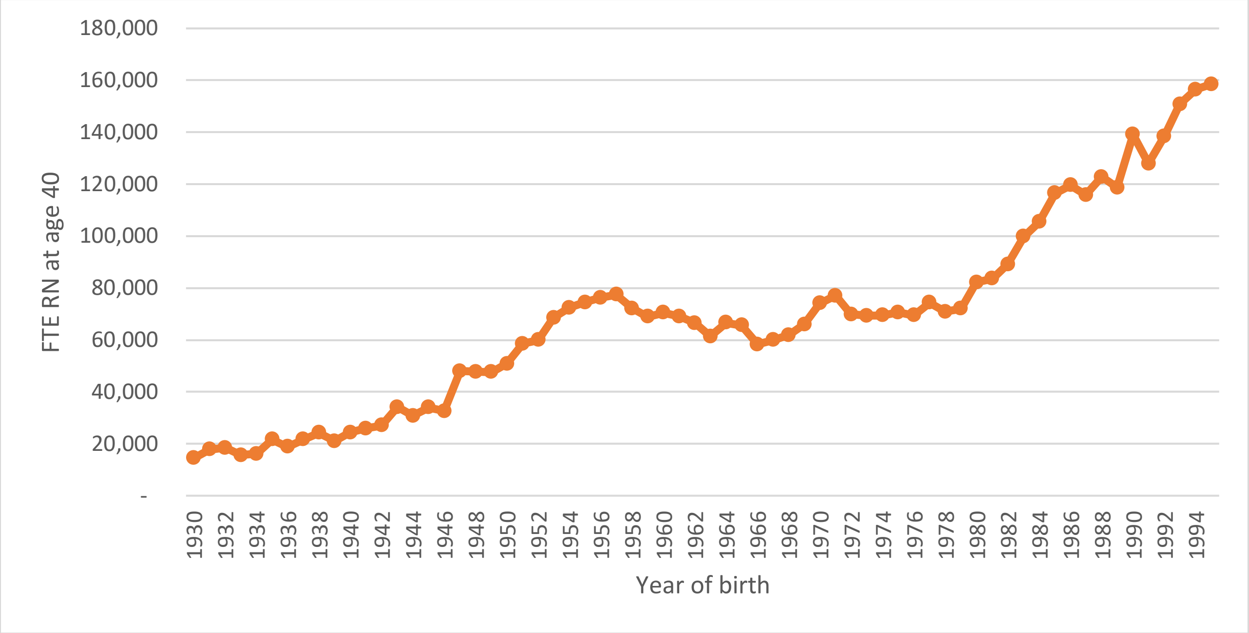

Notes: Data points represent the expected number of FTE RNs produced when the birth cohort is age 40. For example, there were approximately 75,000 RN FTE among RNs born in 1955 when they were age 40 (in 1995). In contrast, there are 120,000 RN FTE expected from the 1986 birth cohort when they are age 40 (in the year, 2026).
Data Source: U.S. Current Population Survey

© 2024 Auerbach DI et al. *JAMA Health Forum.*

eFigure 4. Observed (gray) United States FTE RNs by age group in 2012, forecast FTE RNs by age group in 2022 based on data only through 2012 (blue) and observed FTE RNs by age group in 2022 (yellow)

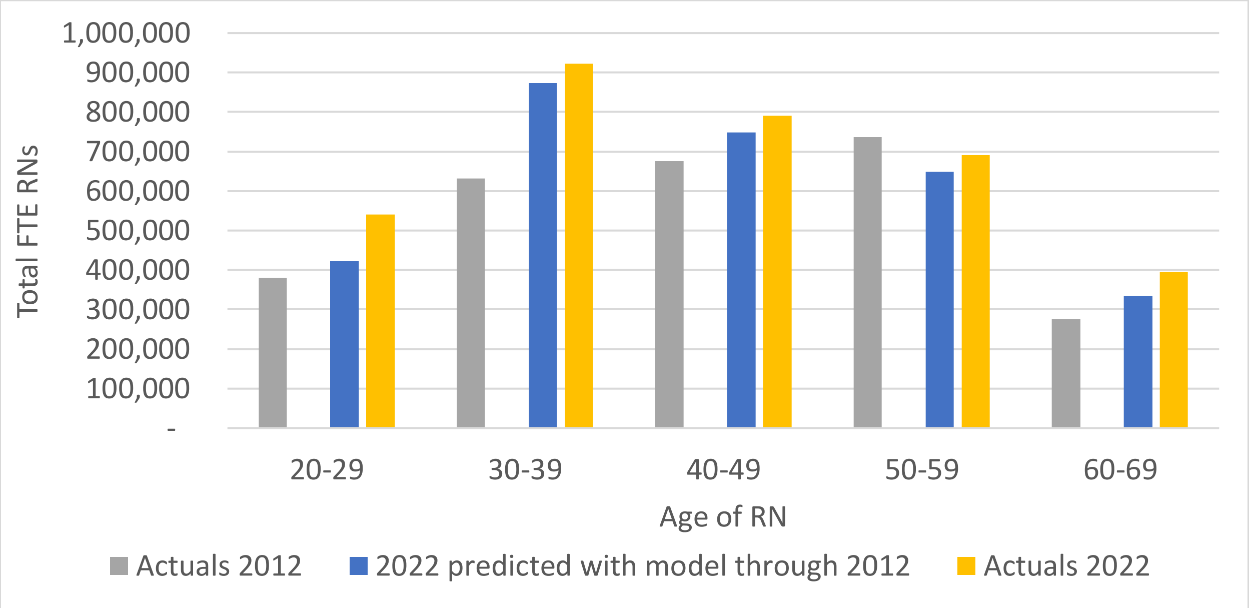

Notes: "Actuals" means observed FTE RNs in the year noted.
Data Source: Forecast model based on data from the U.S. Current Population Survey and other sources noted in Methods.

© 2024 Auerbach DI et al. *JAMA Health Forum.*
