# Supplementary material for: Projecting the Future Registered Nurse Workforce After the COVID-19 Pandemic
Source: JAMA Health Forum. 2024 Feb 16;5(2):e235389. doi: 10.1001/jamahealthforum.2023.5389 (PMC10873770; doi:10.1001/jamahealthforum.2023.5389)
Supplement: Supplement 2. — Data Sharing Statement [file jamahealthforum-e235389-s002.pdf]

Data Sharing Statement

Auerbach. Projecting the Future Registered Nurse Workforce After the COVID-19 Pandemic. *JAMA Health Forum*. Published February 16, 2024. doi:10.1001/jamahealthforum.2023.5389

Data
Data available: Yes
Data types: Data (not involving human participants), Data dictionary
How to access data: https://cps.ipums.org/cps/
When available: beginning date: 10-06-2023

Supporting Documents
Document types: None

Additional Information
Who can access the data: anyone requesting the data
Types of analyses: any purpose
Mechanisms of data availability: without investigator support
Any additional restrictions: One must log in and answer some questions to be able to access data from IPUMS
